# Extending and Matching a High Dynamic Range Image from a Single Image †

**DOI:** 10.3390/s20143950

**Published:** 2020-07-16

**Authors:** Van Luan Tran, Huei-Yung Lin

**Affiliations:** 1 Department of Electrical Engineering, National Chung Cheng University, Chiayi 621, Taiwan; 2 Department of Electrical Engineering, Advanced Institute of Manufacturing with High-Tech Innovation, National Chung Cheng University, Chiayi 621, Taiwan; lin@ee.ccu.edu.tw

**Keywords:** HDR image, extending HDR, LDR2HDR, tone-mapping

## Abstract

Extending the dynamic range can present much richer contrasts and physical information from the traditional low dynamic range (LDR) images. To tackle this, we propose a method to generate a high dynamic range image from a single LDR image. In addition, a technique for the matching between the histogram of a high dynamic range (HDR) image and the original image is introduced. To evaluate the results, we utilize the dynamic range for independent image quality assessment. It recognizes the difference in subtle brightness, which is a significant role in the assessment of novel lighting, rendering, and imaging algorithms. The results show that the picture quality is improved, and the contrast is adjusted. The performance comparison with other methods is carried out using the predicted visibility (HDR-VDP-2). Compared to the results obtained from other techniques, our extended HDR images can present a wider dynamic range with a large difference between light and dark areas.

## 1. Introduction

With the continuous progress of imaging technologies in recent years, one particular demand is to display the acquired images in high quality to resemble the real scenes [1]. The ordinary low dynamic range (LDR) image with 8 bits per channel is usually insufficient to cover all light attributes of a real scene [2]. To derive the brightness information, researchers and practitioners have examined the transformation between the low and high-intensity dynamic extents to obtain the high dynamic range (HDR) images. This has driven the investigation into generating the HDR content from a single LDR image [3,4]. On the other hand, the high dynamic range imaging techniques are also used to overcome some challenging problems in traffic light recognition, lane detection, vehicle, and pedestrian detection at night time for driving assistance [5,6]. The HDR cameras can support more than one channel corresponding to different exposure values to extract the object pattern from a dark background [7]. With high dynamic range imaging, the clear background of the low exposure (dark) channel provides the detection system reliable information. This can make the system more robust when processing the noisy images at the night time [8]. Moreover, stereo vision and high dynamic range imaging can be combined to improve the disparity estimation [9,10].

In recent years, some techniques have been developed for the generation of high dynamic range images from a single input image. It can be applied to mobile imaging devices such as cell phones and compact digital cameras to provide HDR images from an LDR image sensor [11]. They are commonly referred to as the next generation high dynamic range post-processing software [12,13]. HDR imaging in image processing, photography, and computer graphics is a combination of techniques that permits a greater dynamic range of exposure between bright and dark regions. Many proposed methods are based on LDR to HDR expansion. They have been used for HDR compression and enhancing the quality of rendered images with HDR image-based lighting [14]. In [15], Florea et al. presented the utilization of adaptive local luminance to help the algorithm to complete the task of preserving global image details and enhancing local contrasts. However, they did not derive the physical brightness information but focused on adjusting the brightness values in specific image regions such as the foreground areas to create an HDR image.

**Figure 1:**
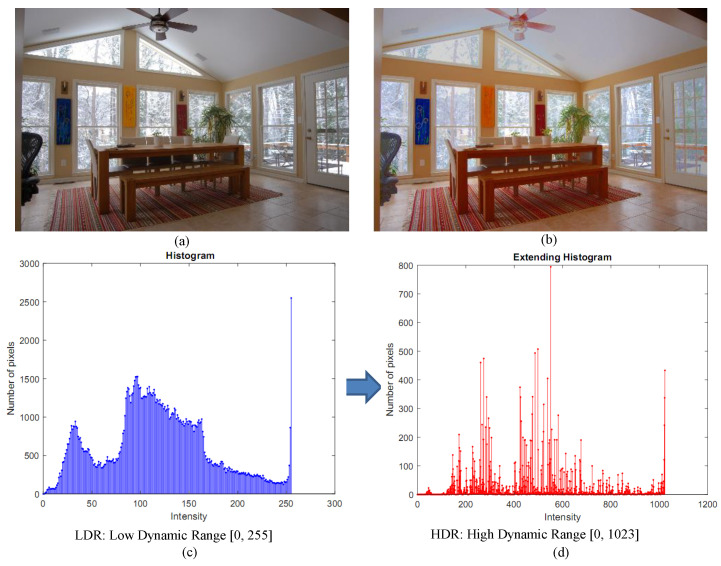
An overview of the proposed method to extend the dynamic range from a single image. (**a**,**c**) A low dynamic range (LDR) input, and the histogram of the intensity image. (**b**,**d**) The tone-mapped high dynamic range (HDR) output and the extended histogram of the intensity image.

To retrieve the hidden information from a single LDR image and create a high-quality perceptual image, we propose a method to generate an HDR image from a single LDR image. As illustrated in Figure 1, the proposed technique aims to improve the image quality and simultaneously balance the dark and bright regions. Firstly, with the separation of intensity and color details, the input LDR color image is converted into the HSI color space, and only the intensity channel is processed. For the brightest regions of the intensity image, we build a combined 2D Gaussian filter. Secondly, the filtered pixel histogram optimized by the integrated 2D Gaussian filter is expanded from 8 bits to 10 bits with a Gaussian distribution in the dynamic range. For a Gaussian distribution, we multiply the histogram, then extend this multiplied strength function and move the histogram bins to the left and right sides until we have an optimal histogram. Thirdly, to obtain an HDR intensity image, the expanded histogram is matched to the input intensity value. We synthesize an HDR intensity image by extending the dynamic range of intensity, which is then combined with the color information to produce an HDR image. Finally, the HDR image is transformed into an LDR image using a globally optimized linear tone-mapping technique to display on normal monitors [16].

The main contributions of this work are as follows:

- We build a hybrid 2D Gaussian filter that is ideal for filtering bright images.
- We propose a method to restore an HDR by a single image and derive a high-quality perceptual image that resembles the real-world scene in dynamic range.
- A solution to match the HDR histogram and the original image using the HDR histogram equalization technique is presented.
- In the proposed system, we include a theoretical analysis and functional assessment of stability and performance.
- Compared with other methods, better results of the tone mapped HDR images, and the VDP-Quality score from our technique are reported.

The rest of this paper is organized as follows. Section 2 provides the literature survey on the related work. Section 3 presents our algorithm of extended dynamic range imaging. Section 4 shows our HDR compression results, the tone mapping results, and the performance evaluation and comparison on dynamic range independent image quality assessment. Finally, Section 5 gives some discussions and concludes this work.

## 2. Related Work

The current HDR image acquisition approaches are mainly divided into two categories– including advanced hardware with a sophisticated image sensor and developing tool with image processing algorithms. The dynamic range image sensor, generally defined by the ratio of the most significant non-saturating output signal to the standard noise deviation under dark conditions, is often considered to be associated with accuracy. The dynamic range of a conventional CMOS image sensor is usually too limited to fulfill the visual signal acquisition criteria [17,18]. With this method, the sensor can work in a maximum dynamic range (120 dB) to obtain good quality HDR images. In [19], Dutton et al. exploited the high dynamic range of the single-photon avalanche diode (SPAD) that is scalable to megapixel arrays. In [20], Martinez et al. proposed a method to optimize the LinLog CMOS sensor and increase its yield for HDR scenes.et al. In CMOS image sensors, all components are required to be included and work in a very small camera system. Thus, the development of the hardware to improve HDR imaging is very challenging.

The new approaches to extend the dynamic range image have attracted the attention of several researchers. Im et al. presented a method to produce three LDR images using local histogram stretching from a single input image. They generate the HDR image by fusing three extended local histograms with noise reduced LDR images. The proposed system is capable of producing ghost HDR images using a single input image [12]. Huo et al. also proposed an innovative approach to traditional dynamic range extension, low dynamic range viewing images for HDR image production and display. The approach allows the image quality is more appropriate for applications such as image segmentation, object detection, and surveillance [4]. Kwon et al. suggested a novel surround diagram, utilizing a single-scale approach to conduct halo reduction and information enhancement. The proposed approach can be easily coupled with modern methods of tone-mapping focused on local surround mapping [21]. The major drawback of LDR to HDR expansion approaches is their failure to extend broad over-exposed regions. This question depends on the scale of the area being over-exposed [22]. Lin et al. proposed an extended dynamic range imaging method by spatially down-sampling the LDR image [23]. Tran et al. presented a method to extend the dynamic range from a single image [24]. The high-resolution information of the input image is converted into the high-quality range information of the output. Results have shown that the extended dynamic range images produced using the proposed method provide improved image quality in terms of visual perception assessment compared with the previous LDR to HDR algorithms.

For deep learning approaches, Lee et al. proposed a deep neural network architecture based on a generative adversarial network (GAN) to solve the inverse tone mapping problem and reconstruct the missing signals from a single LDR image [25]. They used the structural advantages of GAN to infer the natural HDR content extended from a given image. In [26], Eilertsen et al. proposed deep CNNs and hybrid dynamic range autoencoder network for HDR reconstruction. The encoder converts an LDR input to the latent feature representation, and the decoder reconstructs it to an HDR image in the log domain. The reconstructed HDR images from a single exposure are visually convincing, with large improvements in the saturated image regions. However, there is a content-dependent limitation of the network when dealing with the missing information.

For the inverse tone mapping problem, a general framework starts from a single exposure LDR image using the inversion of a tone mapping operators (TMOs) and the expansion map created using the density estimation of light sources [27]. In [28], the inverse gamma and contrast scaling are applied for reverse tone mapping on-the-fly expansion of the dynamic range of legacy. The tone mapped results of the generated HDR images demonstrate a good performance of the tone mapping algorithm [29]. They imitated and deduced the retina response to be locally adaptive, and then estimated the luminance of local adaptation at each pixel in the image. An LDR image and local luminance are applied to the inversed local retina response and reconstruct the original scene’s dynamic range.

## 3. Approach

**Figure 2:**
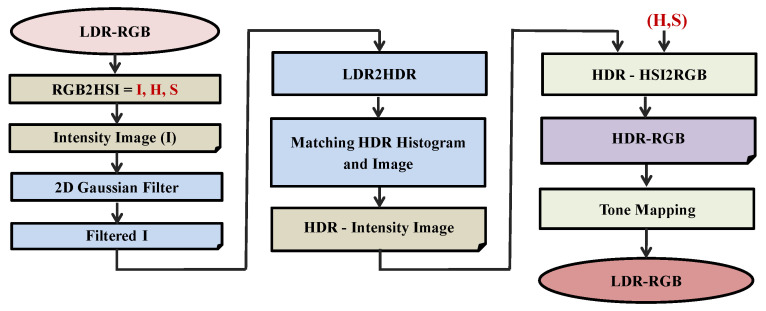
The framework of the proposed LDR2HDR system. The first module is the pre-processing of the intensity image. The second module is to extend the LDR to HDR intensity histogram and match it with the original intensity image to obtain an HDR intensity image. The final module is used to convert the HSI to RGB color spaces for the HDR image, where (H, S) stands for hue and saturation.

The proposed method for HDR image generation consists of three major steps as illustrated in Figure 2. The first step is to design a combined 2D Gaussian filter to reduce the brightest regions. The second step is the procedure to generate a high dynamic range image by extending the histogram to obtain an HDR histogram with a Gaussian distribution. The final step is the tone-mapping for rendering the HDR image on an LDR monitor.

### 3.1. Combined 2D Gaussian Filter

**Figure 3:**
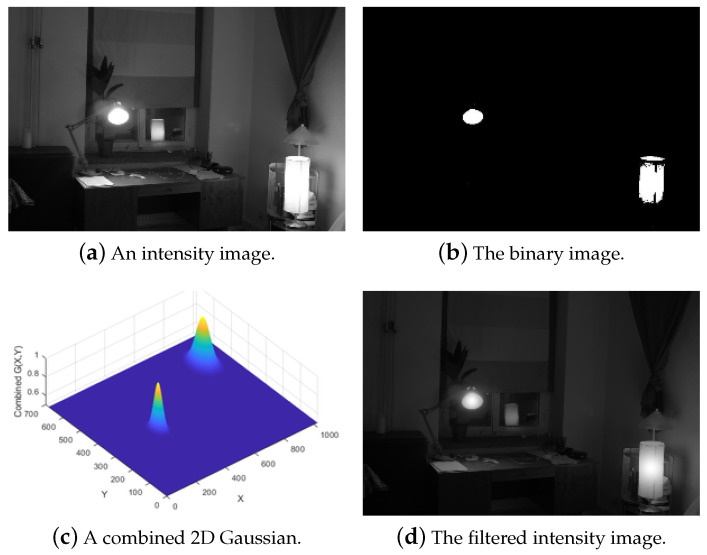
An overview of the filtering of an intensity image with a combined multi-layered 2D Gaussian.

We built a multi-layered combined 2D Gaussian filter. This 2D Gaussian filter is used in the first step to reduce the high-level pixel in the intensity image to balance between the bright regions. Firstly, to produce an intensity image, as shown in Figure 3a, the RGB values of the input image are transformed to the HSI color space. The intensity image is then binarized by applying a threshold to eliminate the low-intensity pixels and retain the high-intensity ones, as presented in Figure 3b. Second, each region in the binary image is organized to a layer, and each layer provides high-intensities in the region. The minimum area of the region is limited by a threshold of 1000 pixels to perform the filtering. Linked components are used to count the blocks in each layer. The 8-connectivity operator is used to define the high-intensity areas. One area is contained in one layer and distributed according to the scale of the high-intensity by a 2D Gaussian. All processed layers are then incorporated to obtain a combined 2D Gaussian, as illustrated in Figure 3c. The 2D Gaussian distribution for each layer is based on the equation

(1) $$G_{2}\left(x_{1},x_{2}\right)=\frac{1}{2\pi \sigma^{2}}\times e^{\frac{-(x_{1}^{2}+x_{2}^{2})}{2\sigma^{2}}}$$

where $x_{1}$ and $x_{2}$ denote the dimension of the region with high intensities, and σ=(v+h)/(2π) where *v*, *h* are the vertical and horizontal size of the region having threshold>250 for the intensity value. The variance of the filter is changed following the regions in the binary image.

As shown in Figure 3b, we have two regions in the binary image. In each layer, one region is applied by a 2D Gaussian filter. Thus, the combined 2D Gaussian filter is used for the combined two layers. This 2D Gaussian filter is designed to reduce the high-intensity values in the image to balance between the bright regions. It is especially for high illumination source areas (such as light, sun). This 2D Gaussian filter is used in some situations when it is required to balance the bright regions in the image. This filter is scaled in the range (0.5;1) as shown in Figure 3c to reduce the high-valued pixels in the filtered intensity image as in Figure 3d.

### 3.2. Extending Dynamic Range

The next task is to produce an HDR image with a high bit depth, say 10-bit. Let filtered-I be a filtered intensity image (see Figure 3d), which is filtered by the combined 2D Gaussian.

We use a filtered image to determine the image histogram and extend the histogram with a Gaussian distribution. The histogram of a filtered image is multiplied with a 1-D Gaussian distribution and then extended. The contrast is also adjusted by the extension and multiplication transformation between the low and high dynamic ranges.

**Figure 4:**
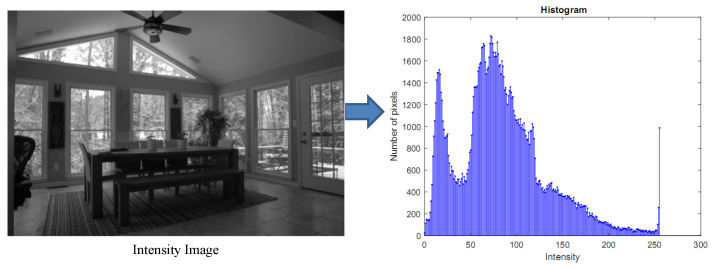
The intensity LDR image and the associated histogram.

**Figure 5:**
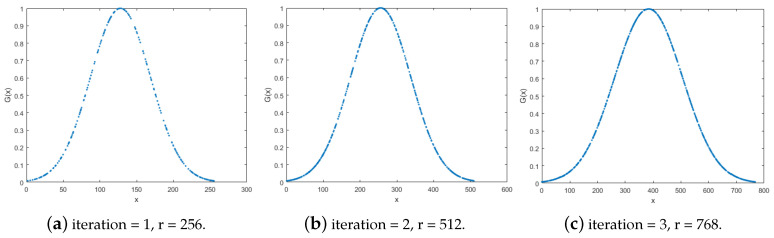
The 1-D Gaussian distribution following the the extending histogram range.

In the first step, we multiply the histogram of filtered image, as shown in Figure 4 with a 1-D Gaussian distribution

(2) $$G_{1}\left(x\right)=\frac{1}{\sqrt{2\pi }\sigma }e^{\frac{-{\left(x-\mu \right)}^{2}}{2\sigma^{2}}}$$

where σ=r/2π is the standard deviation of the distribution, *r* is the extending histogram range, μ=r/2 is the mean, and *x* is a variable of the extending histogram range. Figure 5 shows the 1-D Gaussian distribution following the the extending histogram range.

**Figure 6:**
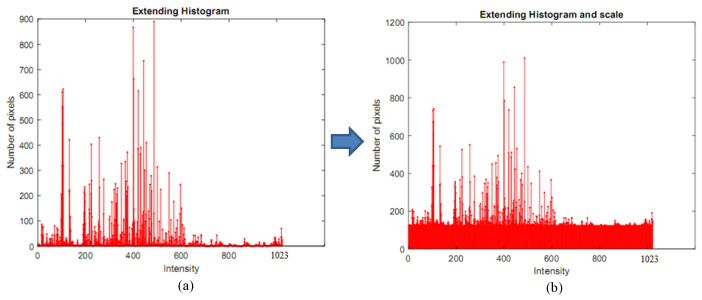
The extended and scaled histogram in the high dynamic range (10 bits). (**a**) An extended histogram from the LDR histogram of the intensity image. (**b**) The scaled histogram with the original image to obtain the same intensity values.

For intensity transformation and extending histogram, we transform the intensity from the left and right to the middle of the histogram by multiplying with 1-D Gaussian distribution. After that, we extend 128 operators for each side with zero extending. The multiplied operators are then shifted for both sides with a step by 2. Thus, we have one zero and nonzero adjacent operator. Afterward, we balance the operator by taking the average of two adjacent operators. Therefore, the extending histogram added 256 operators each time. This iteration repeats until the desired extended histogram is derived, as shown in Figure 6a. The number of iteration is given by

(3) $$i=\frac{2^{n}}{256}-1$$

where *n* is the bit-depth of the HDR image, *i* is the number of extending cycles. The extended histogram is then scaled with the original histogram to obtain the same intensity values, as illustrated in Figure 6b.

**Figure 7:**
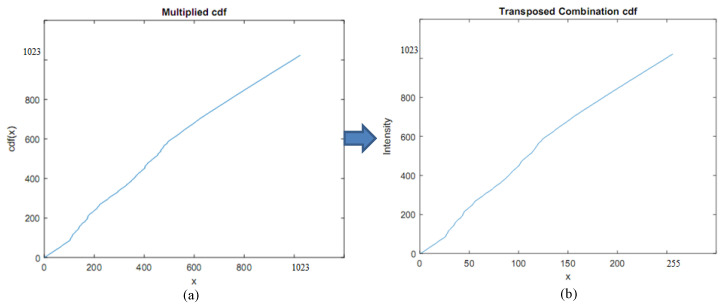
The multiplied cumulative probability of the extended histogram and transposed combination CDF of the extended histogram. (**a**) Multiplied cdf. (**b**) Transposed Combination cdf.

Then, we fit the extended HDR histogram to the LDR intensity image to build an HDR intensity image based on a technique of histogram equalization. Histogram equalization is designed to adjust the intensities of the image to improve the contrast [30]. For a general 8-bit intensity image, the pixel value varies between 0 and 255. Suppose we want to perform histogram equalization on the original image and scale the intensity to the range of [0,1023] to form a 10-bit image. For the extended histogram in HDR, we compute the cumulative probability by the equations

(4) $$pdf\left(X\right)=p\left(R_{k}\right)=\frac{TotalPixelsWithIntensityR_{k}}{TotalPixelsInImageX}$$

and

(5) $$cdf\left(X\right)=\sum_{k=0}^{L-1}p\left(R_{k}\right)$$

where $p(R_{k})$ is the probability of the pixel intensity and *L* is the length of the extended histogram. Finally, cdf(X) needs to be multiplied by *L* and rounded to the nearest integer. The multiplied cumulative probability of the extended histogram is shown in Figure 7.

To match the histogram, we first compute the bin size of the histogram by

(6) $$BinSize=\frac{2^{n}}{256}$$

where *n* is the bit-depth of the image.

Based on Equations (4)–(6), we perform histogram equalization on the original image and scale the intensity to the range of [0,1023] as presented in Equation (7). It is a method to process the intensity image in order to adjust the contrast of the image by modifying the intensity distribution of the extended histogram. The target of this technique is to give a linear trend to the cumulative probability of the extended histogram associated to the image.

(7) $$I_{HDR}\left(u,v\right)\in \left[0,1023\right]\leftarrow T\left(I\left(u,v\right)\in \left[0,255\right]\right)$$

where I(u,v) is the previous intensity of the pixel, $I_{HDR}\left(u,v\right)$ is the new pixel intensity with high dynamic range, *T* is the transformation function of the intensity based on the HDR histogram equalization technique and the merged neighborhood pixels.

**Figure 8:**
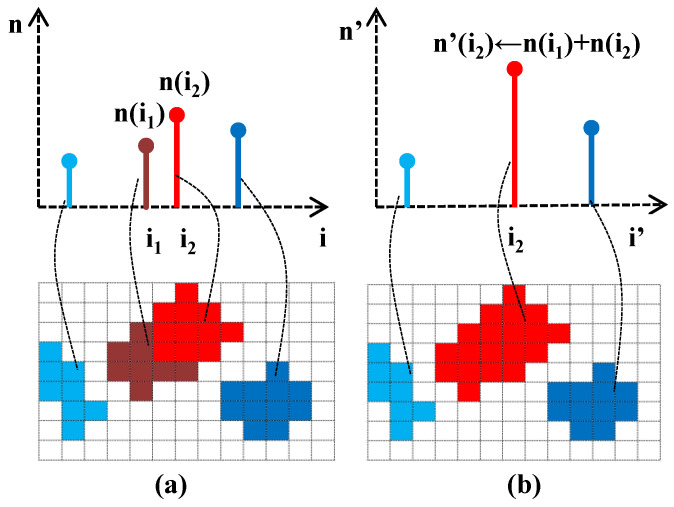
The histogram combining operations of an HDR image. (**a**) An illustration of the bin merge of a histogram. (**b**) The resulting histogram after bin merge.

**Figure 9:**
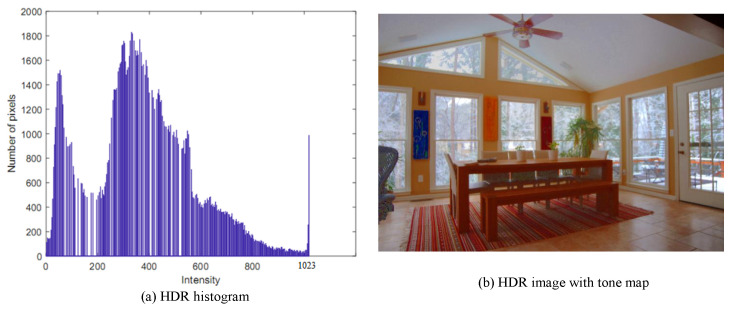
Histogram of HDR intensity image and the HDR image with tone-mapping.

The intensity values and number of pixels corresponding to a given intensity value are adjusted in the extended histogram, and the number of total pixels is unchanged. The new intensity range will be produced with the intensity values of the combined ranges. As illustrated in Figure 8, the neighborhood pixels will be merged into a histogram bin. The number of merge operations depends on the bin size of the histogram. In this research, our goal is to generate a 10-bit HDR image, so the bin size will be set as 1024/256=4. That is, we need to perform the merge operation four times and divide it to BinSize to obtain the combined CDF. It is then transposed to a vector with 256 operations and store the intensity value between 0 and 1023 as shown in Figure 7b. Finally, the transposed combination CDF of the extended histogram is matched with the LDR intensity image. This matching is used to derive an HDR intensity image by finding the indices of nonzero elements and returning the linear indices corresponding to the nonzero entries of the array. We then match all low range values by high range values corresponding to the transposed combination CDF to obtain an HDR intensity image. The HDR intensity image is combined with the hue and saturation images to have an HDR image. The histogram of an HDR intensity image is shown in Figure 9a.

After extending the dynamic range image, we convert the HDR image to an LDR image (HDR2LDR) for display. Due to the limitations of presenting the contrast of HDR images on a normal monitor, the HDR images have to be compressed for display. Tone-mapping is a method for rendering an HDR image to a normal monitor. This method compresses the overall contrast of an HDR image to facilitate the display on LDR devices and produce LDR images with preserved local contrast.

### 3.3. Tone-Mapping

Tone-mapping for HDR images is a technique performed in image processing and computer graphics to map a group of colors to another. It is to approximate the appearance of HDR images in a limited dynamic range and display on a suitable LDR monitor [31]. The tone-mapping techniques still have the problem of strong contrast reduction from the radiance of real scene values while keeping the image details. In general, it is a useful method to display the essential values of an HDR image on the LDR monitors, as shown in Figure 9b. In this work, we use a globally optimized linear windowed tone-mapping method proposed by Shan et al. [16]. It is a high dynamic range compression method that effectively suppresses the global contrast while preserving the local image structure details. This globally non-linear method uses overlapping window-based linear functions to reconstruct the image radiance. The image structures can be preserved even in challenging HDR images that contain either abrupt radiance change or relatively smooth but salient transitions.

## 4. Experiments and Evaluation

**Figure 10:**
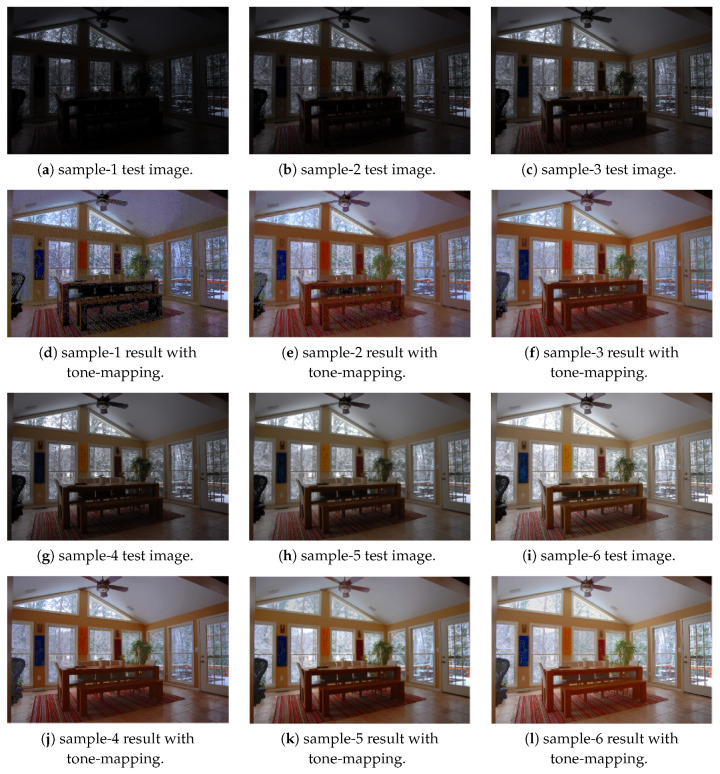
The results of Exposure Sample series images, (**a**–**c**), (**g**–**i**) are the input images, and (**d**–**f**), (**j**–**l**) are the outputs with tone-mapping.

In the experiment we use two sets of images, Window series (1024×683) and Exposure Sample series (484×324), for evaluation [32,33]. Each set consists of LDR images acquired with different exposure settings. In Figure 10, Figure 10a–c,g–i show the input images, and Figure 10d–f,j–l are the results with synthesized tone-mapped images corresponding to the single inputs of Exposure Sample series, respectively. The results demonstrate that our approach effectively preserves the image details for both bright and dark regions and provides high-quality perceptual images.

**Figure 11:**
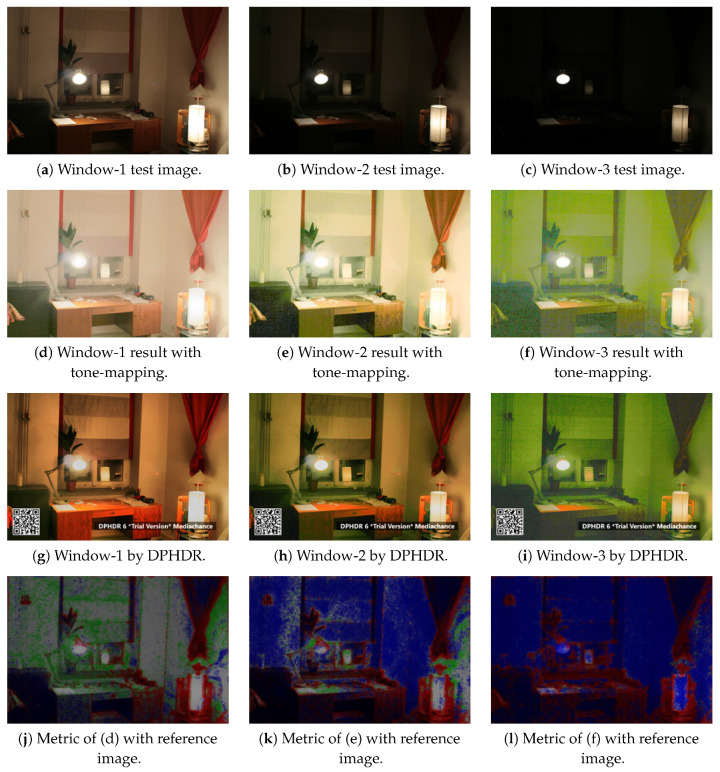
The results of Window series images, (**a**–**c**) are the input images, (**d**–**f**) are the outputs with tone-mapping, (**g**–**i**) are the results from Dynamic Photo HDR-6 for comparison, and (**j**–**l**) are the prediction of the dynamic range independent metric by Image Quality Assessment [35] of the Window series tone-mapping results.

To evaluate the performance of the proposed method, our results are compared with the outputs from Dynamic Photo HDR-6 (DPHDR) [34]. DPHDR is a photography software designed to create HDR images and tone mapped high dynamic range photos. As shown in Figure 11a–c are the input images of the Window series, Figure 11d–f are the results of our method, and Figure 11g–i are the results from Dynamic Photo HDR-6. The visual comparison of the images demonstrates that our method is able to represent a wide range of difference between the bright and dark areas.

**Figure 12:**
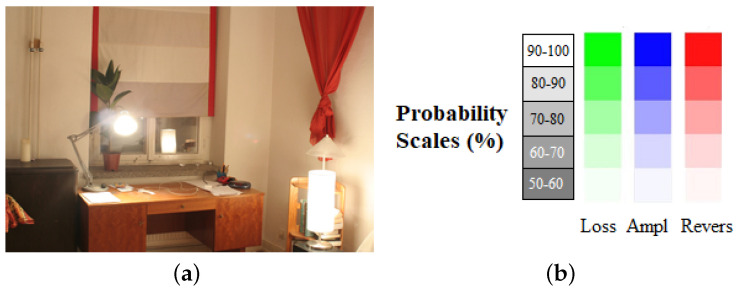
(**a**) The reference image for the prediction of the dynamic range independent metric. (**b**) The probability scale (%) with green for loss of visible contrast, blue for amplification of invisible contrast, and red for reversal of visible contrast for Image Quality Assessment [35].

For objective evaluation, we use a dynamic range independent image quality assessment technique proposed by Aydin et al. [35]. It is designed to compare the images with radically different dynamic ranges by utilizing a model of the human visual system. As shown in Figure 12b, the central idea is a new definition of visible distortion based on the detection and classification of visible changes in the image structure. For the evaluation with a dynamic range independent image quality assessment method, the reference image, as shown in Figure 12a is used.

In Figure 11, we compute the image quality metrics between the results of the tone-mapped Window series and the reference image. Figure 11d–f,j–l show the Window series results with tone-mapping and the quality assessment metric images, respectively. The assessment metric shows that the contrast of the dark regions in our results is increased. It is due to the stretching, which reveals some previously invisible details around the objects in the foreground. The evaluation demonstrates that the information stored in HDR images typically corresponds to the physical values of luminance.

**Figure 13:**
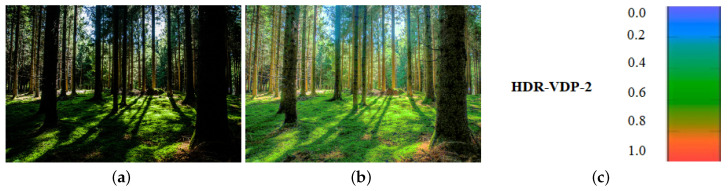
(**a**) An input LDR test image [37]. (**b**) The reference image to predict the visibility difference (**c**) The color scales of the HDR-VDP-2 to show the VDP map.

**Figure 14:**
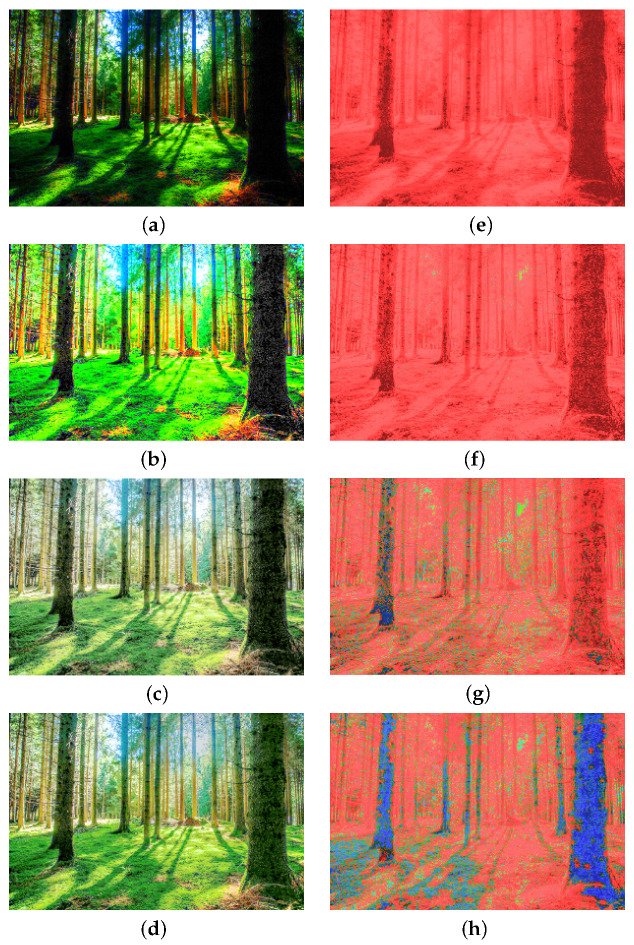
The comparison of our tone-mapped HDR result with those obtained from other methods. The HDR images are evaluated with HDR-VDP-2 [36] to compare and evaluate the VDP-Quality score. (**a**) Huo et al. [38]; (**b**) Masia et al. [39]; (**c**) Endo et al. [37]; (**d**) Ours; (**e**) VDP-Quality score: 55.67; (**f**) VDP-Quality score: 66.32; (**g**) VDP-Quality score: 70.21; (**h**) VDP-Quality score: 73.67.

For more comparison, HDR images are generated using an input LDR image, as shown in Figure 13a. The HDR images are evaluated using HDR-VDP-2 [36] to predict the visibility differences between the tone-mapped HDR images and the reference image (see Figure 13b). HDR-VDP-2 metrics focused on a well-calibrated visual model will accurately estimate the variations in brightness and clarity between pairs of images. The metric is based on a modern visual model for all luminance conditions and is developed from recent measurements of contrast sensitivity. HDR-VDP-2 represents a step towards improved visibility and an indicator of efficiency. The comparison results are shown in Figure 14. Figure 14e–h show the results obtained from HDR-VDP-2. The red-color pixels (close to 1) indicate that we can see the difference from the reference image. In our result, as shown in Figure 14h, it has more blue-color pixels (close to 0) than those obtained from existing methods. This color region demonstrates that we cannot recognize the difference from the reference image. It also indicates that the proposed method approximates the actual scene luminance better than the other methods.

**Figure 15:**
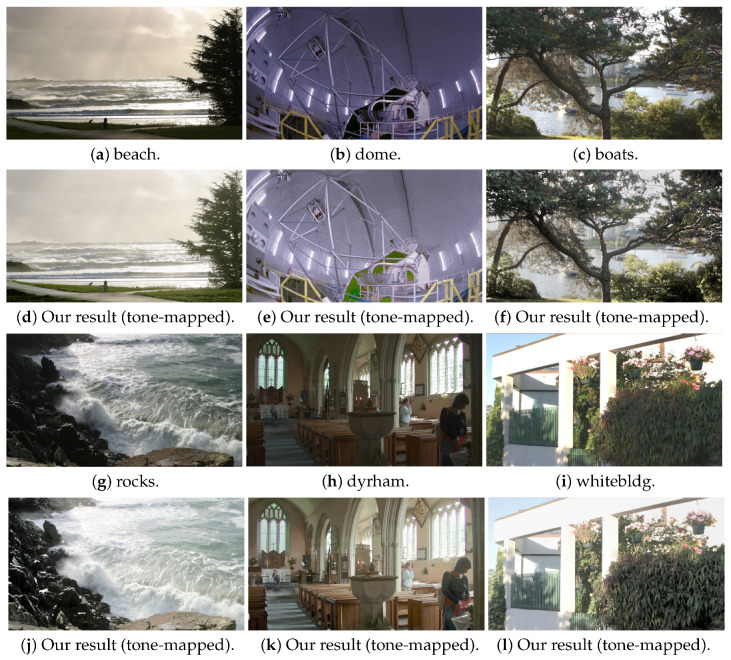
The test images and results using the proposed technique, (**a**–**c**) and (**g**–**i**) are the input images [41], (**d**–**f**) and (**j**–**l**) are the output images with tone-mapping.

Table 1 tabulates the contrast metric evaluation method proposed by Aydin et al. [35]. The test images and results using our approach are shown in Figure 15. In the assessment, contrast loss, contrast amplification, and contrast reverse are used to evaluate the difference between HDR images. Less amplification of invisible contrast means fewer artifacts during dynamic range expansion. The reverse of visible contrast indicates that the difference between the test and reference images is visible. Amplification of invisible contrast implies that the contrast becomes visible in the test images while invisible in the reference images. As shown in Table 1, our results have the smallest total contrast errors in 4 out of 6 images. In terms of contrast reverse, loss, and amplification, the proposed method is better than the techniques by Banterle et al., Huo et al., and Wang et al. Furthermore, our results provide better perceptual quality from contrast analysis of the images presented in Figure 15.

The performance evaluation is carried out for the quantitative comparison with several state-of-the-art HDR prediction techniques. As shown in Table 2, two commonly used image quality metrics, Peak Signal to Noise Ratio (PSNR) and Structural Similarity Index (SSIM), are adopted for the evaluation. A larger PSNR value implies the reconstruction of higher quality and less distortion in the pixel-level. An image with higher SSIM is considered to be more perfect with the contrast quality. We also perform the evaluation with metric HDR-VDP-2, which intuitively reflects human visual perception. The higher HDR-VDP-2 score means the reconstructed HDR image has more renaissance. For the running time comparison, our program and the existing algorithms are tested on a computer with an Intel i7-7700 CPU at 3.6 GHz and 32 GB RAM. An additional NVIDIA 1080 with 8 GB RAM is used for the GPU implementation. The experiment is carried out using the images with a resolution of 1024×683. As shown in Table 3, the whole process takes about 1.47 seconds for our method, which is faster than Huo et al. [38], Masia et al. [39], but slower than Endo et al. [37].

**Figure 16:**
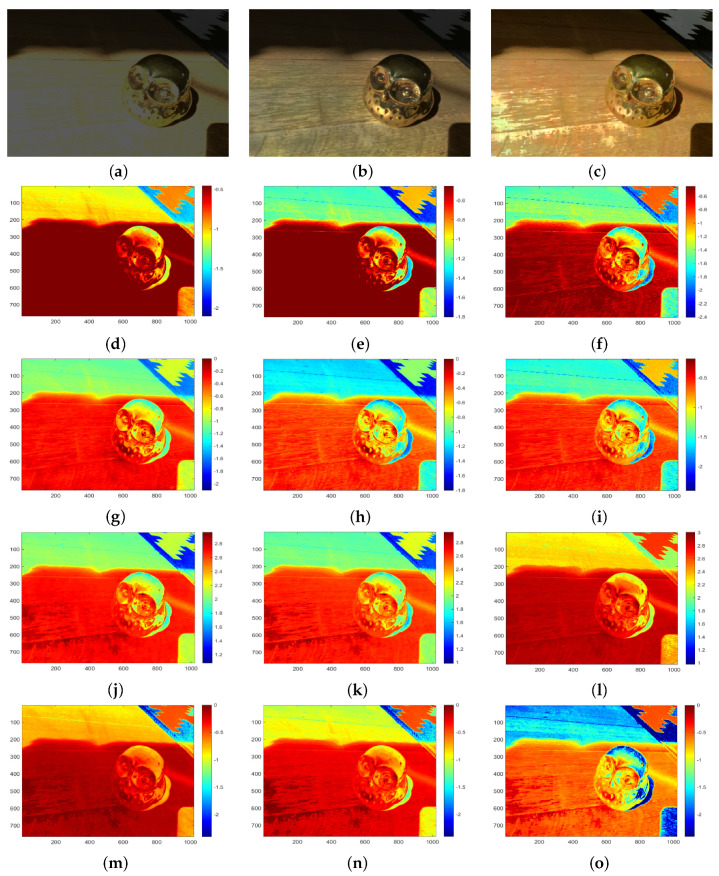
The comparison of our result with those obtained from another method and analyzing the images by separating the red, green, and blue channels by in logarithm (base 10). (**a**) An input test image [26]; (**b**) Eilertsen et al. [26]; (**c**) Ours; (**d**) log10(R) of the test image; (**e**) log10(G) of the test image; (**f**) log10(B) of the test image; (**g**) log10(R) of Eilertsen [26]; (**h**) log10(G) of Eilertsen [26]; (**i**) log10(Blue) of Eilertsen [26]; (**j**) log10(HDR-R) of our result; (**k**) log10(HDR-G) of our result; (**l**) log10(HDR-B) of our result; (**m**) log10(R) of our tone-mapping; (**n**) log10(G) of our tone-mapping; (**o**) log10(B) of our tone-mapping.

As shown in Figure 16, we test our method with an LDR input image to evaluate our HDR and tone-mapping results. We also compare with the output of Eilertsen et al. [26] as presented in Figure 16b. Their method creates an HDR reconstruction from a single exposure by a deep convolutional neural network. Figure 16d–f,g–i show the analysis of Eilertsen’s results by separating the red, green and blue channels in logarithm. Our HDR result is also reported by separating the red, green, and blue channels in logarithm, as shown in Figure 16j–l. Figure 16m–o present the tone-mapping results by separating the three channels for comparison and evaluation. In the tone-mapping result shown in Figure 16c, it can be seen that our method obtains a balanced exposure between the high and low-intensity regions. We also preserve the details of dark and highlight regions. As reported in Figure 16g–o, the quality of reconstruction in each channel using our method has some limitations compared with the results of Eilertsen et al. However, our extending method of the HDR from intensity images is comparable with the HDR reconstruction by a deep convolutional neural network.

## 5. Conclusions

In this paper, we propose a method to generate the HDR and perceptual high-quality image from a single input image. A technique for extending the histogram of an intensity image using a Gaussian distribution is developed. We present an algorithm to merge the HDR histogram with the original image to obtain an HDR image. The experimental results demonstrate that the proposed technique can synthesize more natural images. The results from image quality assessment also illustrate that our method works well and generates high-quality HDR images. The comparison of tone-mapped HDR images and VDP-Quality reports that better quantitative results can be obtained using our approach. In future work, the optimization of our algorithms, and the exploration of more efficient techniques for extending higher dynamic range will be investigated.

## Figures and Tables

**Table 1:** The comparison with the methods by Banterle et al. [27], Huo et al. [40], and Wang et al. [28] using the contrast metric evaluation [35].

	Beach				Dome			
	reverse	loss	amplification	total	reverse	loss	amplification	total
Banterle	8.06	11.63	7.20	26.89	4.87	0.46	23.57	28.90
Huo	4.73	1.32	2.12	8.17	1.12	0.00	18.13	19.25
Wang	5.10	4.22	1.36	10.68	1.39	0.61	5.75	7.75
Ours	2.96	4.93	0.82	10.60	1.56	2.73	2.79	7.08
	**Boats**				**Rocks**			
	reverse	loss	amplification	total	reverse	loss	amplification	total
Banterle	15.07	6.08	11.75	32.90	9.18	5.27	12.23	26.68
Huo	12.03	6.27	0.98	19.28	0.56	0.00	9.24	9.80
Wang	8.19	1.38	0.77	10.34	1.55	0.16	0.17	1.89
Ours	5.69	3.13	1.03	9.85	1.34	0.41	1.23	2.98
	**Dyrham**				**Whitebldg**			
	reverse	loss	amplification	total	reverse	loss	amplification	total
Banterle	7.15	2.50	21.61	31.26	9.74	12.37	4.61	26.72
Huo	9.73	10.89	0.86	21.48	13.30	14.94	3.31	31.55
Wang	6.12	3.28	0.59	9.99	10.58	2.60	3.49	16.67
Ours	4.23	3.46	0.68	8.37	7.56	3.78	2.43	13.77

**Table 2:** The comparison with the start-of-the-art HDR prediction methods using different metrics.

Method	PSNR	SSIM	HDR-VDP-2
Huo et al. [38]	14.86	0.63	55.67
Masia et al. [39]	18.90	0.72	66.32
Endo et al. [37]	28.58	0.95	70.21
Ours	27.95	0.93	73.67

**Table 3:** The processing time of the proposed technique and other methods.

Method	Huo et al. [38]	Masia et al. [39]	Endo et al. [37]	Ours
Processing time [s]	1.92	2.15	0.56	1.47
